# Optical coherence tomography angiography as a surrogate marker for end-organ resuscitation in sepsis: A review

**DOI:** 10.3389/fmed.2022.1023062

**Published:** 2022-10-20

**Authors:** Ella Courtie, Ahmed Gilani, Tonny Veenith, Richard J. Blanch

**Affiliations:** ^1^Neuroscience and Ophthalmology Research Group, University of Birmingham, Birmingham, United Kingdom; ^2^Department of Ophthalmology, Queen Elizabeth Hospital Birmingham, University Hospitals Birmingham NHS Foundation Trust, Birmingham, United Kingdom; ^3^Surgical Reconstruction and Microbiology Research Centre, University Hospitals Birmingham NHS Foundation Trust, Birmingham, United Kingdom; ^4^Critical Care Unit, Queen Elizabeth Hospital Birmingham, University Hospitals Birmingham NHS Foundation Trust, Birmingham, United Kingdom; ^5^Institute of Inflammation and Ageing, University of Birmingham, Birmingham, United Kingdom; ^6^Department of Trauma Sciences, University of Birmingham, Birmingham, United Kingdom; ^7^Academic Department of Military Surgery and Trauma, Royal Centre for Defence Medicine, Birmingham, United Kingdom

**Keywords:** optical coherence tomography angiography, OCTA, sepsis, critical illness, retinal blood flow

## Abstract

Sepsis is a severe illness which results in alterations in the end organ microvascular haemodynamics and is associated with a high risk of mortality. There is currently no real-time method of monitoring microcirculatory perfusion during sepsis. Retinal microcirculation is closely linked to cerebral perfusion and may reflect systemic vascular alterations. Retinal perfusion can be assessed using the non-invasive imaging technique of optical coherence tomography angiography (OCTA). This narrative review aims to discuss the utility of using retinal imaging and OCTA in systemic illness and sepsis. OCTA can be used as a functional, non-invasive and real-time biomarker along with other haemodynamic parameters for assessing and managing patients with sepsis.

## Introduction

Sepsis is a dysregulated response to infection causing widespread inflammation, leading to life-threatening organ dysfunction (1). Estimates suggest that 147,000 individuals (10,000 of which are children) are admitted with sepsis annually in the UK, with sepsis killing at least 46,000 people each year (2). Sepsis can progress to septic shock with high mortality and is associated with profound circulatory and metabolic abnormalities (1), resulting in multiple organ failure (3, 4) if not intervened early.

No single diagnostic test can definitively establish the diagnosis of sepsis or septic shock. Diagnosis is clinical and relies on early recognition of hypotension, high cardiac output, and end-organ damage. The quick Sequential Organ Failure Assessment (qSOFA) score is a fast scoring method to identify patients with suspected infection who are likely to die in hospital, and which takes into consideration respiratory rate, altered mentation, and systolic blood pressure (BP) (1). The SOFA score is an expansion of the qSOFA and is used for patients with suspected infection within the intensive therapy unit (ITU) to determine the extent of the end-organ failure and mortality associated with sepsis (1).

Altered microvascular blood flow is a core feature of sepsis (5–7), involving reduced functional capillary density and faulty capillary perfusion, ultimately reducing the amount of oxygen delivered to essential organs, causing organ dysfunction and critical organ failure (3, 8).

Fluid resuscitation is a standard intervention to treat septic patients and prevent end-organ dysfunction, such as renal failure, which is a significant cause of sepsis-induced morbidity and mortality (9). However, using inotropes and fluids has variable results (10).

There is currently no real-time method to monitor microcirculatory reperfusion in septic patients, which would be beneficial for monitoring sepsis progression and response to therapeutic intervention. This review discusses the potential for retinal blood flow as a surrogate marker for end-organ (brain) perfusion in sepsis.

## Retinal and cerebral perfusion relationship

The retinal vasculature arises directly from the cerebral blood vessels, as the internal carotid artery supplies the ophthalmic artery, which supplies the central retinal artery: the sole blood supply to the retina (11). Both retinal and cerebral blood flow depends on the balance between arterial perfusion pressure and resistance from vascular beds (12, 13), with vasculature in the eyes and brain responding to local metabolic factors to maintain ocular and cerebral perfusion pressure, respectively (14). Due to shared vessel anatomy and similarities in microcirculatory regulation, changes in retinal perfusion correlate with cerebral microcirculation in healthy patients and may do so in systemic diseases (14). The retina and brain showed similar blood flow changes in animal models of hemorrhagic shock and hypercapnia models (15–17). In healthy human subjects, retinal blood flow is affected similarly to cerebral blood flow by nimodipine (18).

## Optical coherence tomography and angiography

**FIGURE 1:**
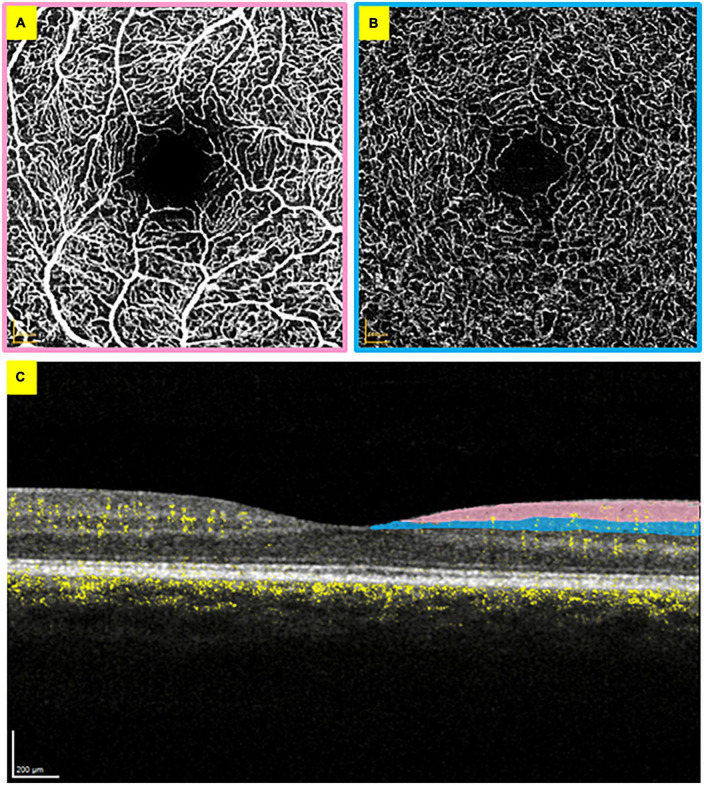
Optical coherence tomography angiography scans from the right eye. **(A)** *En face* superficial vascular plexus layer with the foveal avascular zone visible in the centre. **(B)** *En face* intermediate capillary plexus layer. **(C)** Cross-section of the retina, with blood flow visible in yellow. The added colour areas correspond to the borders of panels **(A,B)** to demonstrate the vascular bed locations.

Optical coherence tomography (OCT) and OCT angiography (OCTA) are non-invasive imaging techniques that allow high-resolution cross-sectional retinal imaging of its structure and blood flow respectively (19, 20), without an injection of contrast (21). OCTA can capture each distinct vascular bed of the retina, including the superficial vascular plexus, composed of larger arteries/arterioles and veins/venules supplied by the central retinal artery; and the intermediate capillary plexus (Figure 1) (22). OCT and OCTA scans are based on the estimation of light reflectivity and blood flow (23). The scans quickly acquire and offer instantaneous qualitative (and quantitative) data in some devices. Previous studies have shown the feasibility (24), stability (25) and optimal analysis methods for using OCT and OCTA in a critical care setting, suggesting these scans may offer sensitive and reproducible means to study retinal manifestations of systemic disease.

## Methods to monitor sepsis

Sepsis is a time sensitive critical illness, with early recognition and regular monitoring of progression proving pivotal to survival, especially when there may be haemodynamic instability (1). It is therefore important to have precise haemodynamic recording and analysis to ensure that the supportive interventions are adequate to improve outcomes. Myocardial and peripheral vascular dysfunction are equally important in sepsis, with functional assessment of cardiovascular reserve and vessel perfusion necessary to ensure that end-organ blood flow is maintained, ultimately preventing critical organ failure (26).

Monitoring mean arterial pressure (MAP) is a standard assessment in sepsis as it approximates organ perfusion pressure, with a MAP of <70 mmHg deemed a criteria point for sepsis (1). Cardiac output is also monitored during sepsis and acts as an indicator of response to treatment (26). Cardiac output measurements such as pulmonary artery catheter, aortic velocimetry, arterial waveform analysis and trans oesophageal echocardiography do not measure or estimate microcirculatory dysfunction in the brain (27). Markers of tissue perfusion such as mixed venous oxygen saturation and lactate estimate global hypoperfusion and not cerebral microcirculation (26).

## Systemic disturbances and retinal blood flow in non-septic systemic diseases

Retinal blood flow has been investigated in many disease states which affect different organ systems, suggesting its potential for use as a biomarker across numerous diseases. The following study results are detailed in Table 1.

**TABLE 1:** Summary of studies investigating retinal blood flow changes in disease states.

Authors	Study groups (*n*)—human unless specified otherwise	Imaging technique	Metrics investigated	Results
Chua et al. (28)	Systemic hypertension (77)	OCTA (AngioVue; Optovue, Inc., Fremont, CA, USA)	Capillary density	Reduced capillary density in hypertensive patients with poorly controlled BP (148 ± 8 mmHg) compared with patients with well controlled BP (125 ± 9 mmHg)—27.2 vs. 34.7%, *p* = 0.039.
Terheyden et al. (29)	Hypertensive crisis (17) vs. healthy controls (18)	OCTA (Zeiss PLEX Elite 9000; Carl Zeiss Meditec, Dublin, CA, USA)	VD SVD	Reduced VD in hypertensive patients (systolic BP ≥ 180 mmHg or diastolic BP ≥ 110 mmHg) compared with healthy controls—0.086 vs. 0.103, *p* = 0.004. Reduced SVD in hypertensive patients compared with controls—4.7 × 10^–8^ vs. 5.2 × 10^–8^, *p* = 0.002.
Lange et al. (30)	AF (34) vs. healthy controls (35)	OCTA (RTVue XR Avanti with AngioVue, Optovue Inc, Fremont, CA, USA)	Flow density	Prior to treatment, reduced flow density in superficial vasculature in patients with AF compared with controls—48.77 vs. 53.01%, *p* = 0.000, and in deep vasculature—55.61 vs. 57.15%, *p* = 0.005. Following treatment in patients, flow density in the optic nerve head improved—51.82 vs. 52.49%, *p* = 0.007.
Kannenkeril et al. (31)	CKD (96)	Fundus photographs (Canon CR-2^®^ camera, Ôta, Tokyo, Japan)	CRAE and CRVE	Reduced CRAE and CRVE in stage 3 CKD patients compared with stage 2 patients—CRAE: 130.5 vs. 141.1 μm, *p* = 0.030; CRVE: 206.0 vs. 220.8 μm, *p* = 0.004.
Coppolino et al. (32)	Intradialytic hypotension (35)	OCTA (Optovue, Fremont, CA, USA)	FAZ area VD	Patients who experienced hypotensive episodes following dialysis had pre-dialysis measures showing reduced FAZ area (*p* = 0.03) and higher VD in the superficial and deep vascular layers (*p* = 0.02–0.04).
Guzinger et al. (33)	TAVI (28)	OCTA (Plex Elite 9000, Carl Zeiss Meditec AG, Jena, Germany)	Retinal capillary dropout VD FAZ size	Following TAVI, 28.6% of patients showed new retinal capillary dropout in the superficial and deep capillary layers. There was no change in VD or FAZ size between pre- and post-TAVI scans.
Yang et al. (34)	Sjögren syndrome (12) vs. healthy controls (12)	OCTA (Optovue, Fremont, CA, USA)	VD	Reduced VD in patients with Sjögren syndrome compared with healthy controls in the superficial and deep vascular layers (*p* < 0.05).
Songur et al. (35)	COPD (70) vs. healthy controls (71)	OCTA (RTVue XR Avanti System; Optovue Inc, Fremont, CA, USA)	VD FAZ diameter pO_2_	Reduced VD in COPD patients compared with healthy controls in both the superficial and deep capillary layers—superficial: 46.46 vs. 48.81%, *p* = 0.002; deep: 46.24 vs. 49.54%, *p* = 0.002. FAZ diameter was increased in COPD patients—0.28 vs. 0.23, *p* = 0.002. There was a moderate correlation between pO_2_ and VD in the superficial and deep layers (*r* = 0.559/0.800 respectively), and a negative correlation between pO_2_ and FAZ diameter (*r* = -0.876).
Vaes et al. (36)	COPD (246)	Fundus imaging (device not specified)	CRAE and CRVE	In patients with COPD, older age, higher systolic BP and lower levels of systemic inflammation were associated with narrower retinal venules (-0.654 μm; -0.229 μm; and -13.767 μm, *p* < 0.05 respectively). Older age and higher systolic BP were associated with narrower retinal arterioles (-0.224 μm, *p* = 0.042; -0.136 μm, *p* < 0.001).
Erikkson et al. (38)	Sepsis (31)	Fluorescein angiography imaging (HRA 2-00153; Heidelberg Engineering, Heidelberg, Germany)	Retinal arterial filling time (RAFT)	Septic patients with prolonged RAFT (>8.3 s) had a higher intraocular pressure (17.8 vs. 14 mmHg, *p* = 0.029); lower cardiac index (2.1 vs. 3.1, *p* = 0.039); and lower levels of C-reactive protein (139 vs. 254 mg/L, *p* = 0.011) than patients with a short RAFT (<8.3 s).
Simkiene et al. (39)	Sepsis (40) vs. healthy controls (20)	Fundus photography (Optomed Oy, Finland)	CRAE and CRVE Vascular length density	Median CRAE was higher and vascular length density was lower in septic patients compared with healthy controls—165 vs. 146 μm, *p* = 0.002; 0.51 vs. 0.64%, *p* < 0.001 respectively. CRVE did not differ between groups. There was a correlation between CRVE and lactate levels (*p* = 0.005), arterial tortuosity and diastolic BP (*p* = 0.036), vascular length density and lactate (*p* = 0.047), and vascular length density and cardiac index (*p* = 0.014).
Simkiene et al. (40)	Sepsis (48) vs. healthy controls (20), and sepsis survivors (19) vs. non-survivors (29)	Incident dark-field imaging (Braedius Medical, Huizen, Netherlands)	Conjunctival perfused VD, proportion of perfused vessels, and microvascular flow index. Retinal vascular length density	There were significant reductions in all conjunctival measures in septic survivors and non-survivors compared with healthy controls: total VD: 11.5 vs. 16.2 mm/mm^2^; perfused VD: 9.4 vs. 16.1 mm/mm^2^; proportion of perfused vessels: 86.8 vs. 99.3%; microvascular flow index: 2.42 vs. 3.00. There was lower perfused VD in non-survivors compared with survivors at 24 h (8.3 vs. 10.9 mm/mm^2^, *p* < 0.05). Non-survivors had lower retinal vascular length density at 6 and 24 h compared with baseline (0.43, 0.46, 0.50% respectively, *p* < 0.05).
Parisi et al. (41)	Pneumonia caused by COVID-19 (63) vs. healthy controls (45)	OCTA (DRI-OCT Triton SS-OCT Angio, Topcon Inc., Tokyo, Japan)	FAZ area VD	Larger FAZ area in patients with pneumonia compared with healthy controls in superficial and deep vascular layers—326.81 vs. 251.03 μm^2^, *p* = 0.000; 357.76 vs. 237.12 μm^2^, *p* = 0.000 respectively, Reduced VD in the foveal area of the choriocapillaris in pneumonia patients (51.42 vs. 52.43%, *p* = 0.046), but no difference between groups in VD of superficial or deep vascular layers.
Park et al. (42)	Septic rat model (5) Haemorrhagic shock rat model (5)	OCTA (device not specified)	BFI	BFI reduced in the retina from baseline to 95.1% at 6 h after induction, with choroidal BFI reducing to 87.4% from baseline. BFI reductions happened in parallel with decreasing MAP (100.7–54 mmHg) and increasing lactate (0.77–2.65 mmol/L). In the haemorrhagic shock model, retinal BFI reduced to 97.7% following onset while choroidal BFI reduced to 95.1%. Following resuscitation, retinal and choroidal BFI increased to 101.6 and 100.5% respectively, with choroidal ultimately settling on 96.5%. MAP decreased during shock to 64 mmHg and increased to 90.2 mmHg following resuscitation, but ultimately reduced to 72.4 mmHg.
Alnawaiseh et al. (43)	Haemorrhagic shock ovine model (5)	OCTA (Optovue Inc., Fremont, CA, USA)	Retinal flow density Conjunctival proportion of perfused vessels Conjunctival microvascular flow index	Flow density reduced during shock from 44.7 to 34.5% (*p* = 0.027), returning to baseline levels following resuscitation at 46.9% (*p* = 0.027). The proportion of perfused vessels in the conjunctiva also reduced during shock from 100 to 72% (*p* = 0.012) and increased to 98.7% following resuscitation (*p* = 0.173). Flow index also decreased from 3.1 to 1.9 (*p* = 0.034) and increased following resuscitation (*p* = 0.081). Both retinal and conjunctival microvascular changes occurred in correlation with each other and haemodynamic measures during shock, with MAP decreasing from 117 to 33 mmHg (*p* = 0.001), cardiac index reducing from 2.5 to 1.1 L/min/m^2^ (*p* = 0.051), and central venous oxygen decreased from 86.5 to 38.8% (*p* = 0.003).

OCTA, optical coherence tomography angiography; BP, blood pressure; VD, vessel density; SVD, skeletonised vessel density; AF, atrial fibrillation; CKD, chronic kidney disease; CRAE, central retinal arteriolar equivalent; CRVE, central retinal venular equivalent; FAZ, foveal avascular zone; TAVI, transcatheter aortic valve implantation; COPD, chronic obstructive pulmonary disorder; pO_2_, partial pressure of oxygen; BFI, blood flow index.

### Acute haemodynamic disturbance

In a study involving patients with systemic hypertension, OCTA showed that patients with poorly controlled BP (148 ± 8 mmHg) had a significantly lower retinal capillary density in the deep vascular plexus than patients with well-controlled BP (125 ± 9 mmHg) (*p* = 0.039) (28). Similar findings have been shown in patients with hypertensive crisis (systolic BP ≥ 180 mmHg or diastolic BP ≥ 110 mmHg), with retinal deep vasculature vessel density (VD) and skeletonised VD from OCTA scans significantly reduced in patients compared with healthy controls (*p* = 0.004 and 0.002 respectively) (29). Patients with atrial fibrillation (AF) had significantly lower retinal flow density in the superficial and deep vasculature compared with healthy controls (*p* = 0.000 and 0.005 respectively) before pulmonary vein isolation treatment. After treatment, only flow density in the optic nerve head significantly improved (*p* = 0.007), showing that retinal perfusion was reduced in patients with AF, with OCTA offering novel information on global perfusion in AF patients (30).

### Chronic haemodynamic disturbance

Patients with chronic kidney disease (CKD) have differing retinal microvascular calibres depending on the disease stage (31). Using digital retinal imaging from fundus photographs, central retinal arteriolar or venular equivalent (CRAE or CRVE) were significantly lower in patients with stage 3 CKD than those with stage 2 CKD (*p* = 0.004), showing retinal vascular thinning in proportion to CKD severity, suggesting retinal vessel calibre may serve as a biomarker of CKD progression (31). Patients who undergo haemodialysis with hypotensive episodes in the 30 days after dialysis, had pre-dialysis measures showing a reduced foveal avascular zone (FAZ) area (*p* = 0.03), and a higher VD of the superficial and deep capillary plexus (*p* = 0.002–0.04), indicating higher retinal blood flow in these patients, compared with those who did not have hypotensive episodes (32). This suggests a single OCTA measurement may be able to stratify the risk of intradialytic hypotension.

After transcatheter aortic valve implantation, OCTA scans found new retinal capillary dropout in 28.6% of participants in both the superficial and deep capillary plexi (33), while vessel density and FAZ size remained stable between pre- and post-implantation scans, with authors suggesting lesions were too small to have significant overall blood flow effects (33).

Patients with Sjögren syndrome—a chronic systemic autoimmune disease—had reduced vessel density in the superficial and deep microvasculature (*p* < 0.05) compared with healthy controls, although as they only included 12 patients, a larger sample would be required to quantify vascular changes with disease progression (34).

Patients with chronic obstructive pulmonary disorder (COPD) had reduced retinal vascular density compared with healthy controls in the superficial and deep capillary plexi on OCTA (*p* = 0.002) (35). COPD patients also had a larger FAZ diameter than healthy participants (*p* = 0.002) (35), and there was a moderate correlation between partial pressure of oxygen (pO_2_) and superficial/deep vascular density (*r* = 0.559/0.800 respectively), and a highly negative correlation between pO_2_ and FAZ size (*r* = −0.876) (35), suggesting that retinal vascular density by OCTA may be useful to monitor disease progression in COPD patients (35). Retinal imaging in COPD has also shown that older age, higher systolic BP and lower levels of systemic inflammation are associated with narrower retinal venules (*p* < 0.05) (36).

These studies highlight the increasing use of retinal vasculature to investigate disease states, progression, and treatment response.

## Sepsis, the retina, and haemodynamics

As cerebral and retinal perfusion are closely related, and sepsis primarily affects that microcirculation, the retina is expected to reflect disease progression during a septic episode. The following study results are detailed in Table 1.

Fluorescein angiography (FA) is a retinal imaging technique in which fluorescein dye is injected intravenously to visualise the superficial retinal vasculature (37). Thirty-one septic patients in ITU underwent FA to determine how retinal arterial filling time (RAFT) compared with macrohaemodynamic status (38). Patients with prolonged RAFT (>8.3 s) had a higher intraocular pressure (*p* = 0.029); lower cardiac index (CI) (*p* = 0.039); and lower levels of C-reactive protein (*p* = 0.011) than patients with a short RAFT (<8.3 s), although there were no differences when considering vasopressor dose, MAPs, or serum lactate levels (38). Septic patients with a prolonged RAFT, therefore, had an impaired inflammatory response compared with those who had a short RAFT, demonstrating the potential to indicate macro- and microcirculatory defects (38). Although FA is an invasive technique and carries risk of an adverse reaction to the fluorescein dye, the demonstration of FA showing microcirculatory defects further adds to the evidence that retinal perfusion may reflect systemic perfusion, especially as RAFT corresponded with haemodynamic measures.

Using a handheld fundus photography device, large retinal vessel calibres and density were calculated by CRAE and CRVE respectively, and in septic patients, the median CRAE was higher and vascular length density lower compared with healthy controls (*p* = 0.002 and *p* < 0.001 respectively), yet CRVE did not differ between the two groups (*p* = 0.396) (39). However, there was a significant correlation between CRVE and lactate levels (*p* = 0.005), arterial tortuosity and diastolic BP (*p* = 0.036), vascular length density and lactate (*p* = 0.047), and vascular length density and CI (*p* = 0.014). This use of fundus imaging—a non-invasive technique—showing retinal vascular changes in septic patients demonstrates the potential to use similar retinal imaging techniques in this cohort of patients. A strength of this technique is its ability to quantify the microvascular dysfunction reproducibly.

Incident dark field (IDF) imaging—a non-invasive technique using a green light source absorbed by haemoglobin to detect red blood cells—has been used to assess conjunctival microvascular blood flow (40). When comparing septic patients with healthy controls, and septic survivors against non-survivors, there were significant reductions (*p* < 0.001) in all IDF conjunctival microcirculation measures in septic patients compared with healthy controls. There was also significantly lower perfused VD in non-survivors compared with survivors at 24 h (*p* < 0.05). Non-survivors also had significantly lower retinal vascular length density (large vessels) by fundus photography at 6 and 24 h compared with baseline (*p* < 0.05) (40). This correlation between conjunctival and retinal flow changes in septic patients suggests the link between peripheral (conjunctival) and central (retinal) blood flow reduction during sepsis.

In patients with bilateral pneumonia caused by COVID-19 (41), OCTA scans showed FAZ area was significantly larger in COVID-19 patients in the superficial and deep vascular layers (*p* = 0.000) compared to healthy controls. VD was also reduced in the foveal area of the choriocapillaris (*p* = 0.046). However there was no difference between groups in either the superficial or deep plexus VD percentage (41).

## Retinal and conjunctival blood flow in sepsis/haemorrhagic shock resuscitation

While the previous studies have shown that retinal perfusion is altered in septic patients compared with healthy controls, it is important to investigate whether reduced retinal blood flow returns with resuscitation and treatment of sepsis, how this correlates with haemodynamic measures, and if this can be detected using non-invasive retinal imaging. The following study results are detailed in Table 1.

Researchers using a septic rat model calculated blood flow index (BFI) from retinal and choroidal OCTA scans at 6 and 24 h after induction (42) which showed both retinal and choroidal BFI reduced during shock which occurred in parallel with a decrease in MAP and an increase in blood lactate. In a rat haemorrhagic shock model, retinal and choroidal BFI reduced during shock and increased after resuscitation. MAP also decreased during shock and increased following resuscitation (42). This demonstrates that retinal blood flow may reflect systemic effects from haemorrhagic shock as it alters dynamically in correlation with haemodynamic markers, and that OCTA can detect these changes.

In an ovine model of haemorrhagic shock, MAP decreased significantly (*p* = 0.001) after indution and then increased close to baseline levels following resuscitation (*p* = 0.164). CI also decreased during shock (*p* = 0.051) and increased after resuscitation (*p* = 0.001), and central venous oxygen decreased during shock (*p* = 0.003) and increased to baseline levels after resuscitation (*p* = 0.027) (43). In the retina, OCTA flow density reduced during shock (*p* = 0.027) and returned to baseline levels following resuscitation (*p* = 0.027). Conjunctival flow also showed similar changes, with the proportion of perfused vessels reducing significantly during shock compared with baseline (*p* = 0.013), increasing following resuscitation to near baseline (*p* = 0.173). Microvascular flow index also decreased (*p* = 0.034) and then increased following resuscitation (*p* = 0.081). Further, the authors showed that flow density in the superficial retinal OCTA significantly correlated with changes to conjunctival microcirculation and haemodynamic measures (*p* = 0.002–0.014) (43). The changes to conjunctival blood flow demonstrate that conjunctival and retinal microcirculation may be able to reflect changes to systemic circulation during shock and therefore offer a non-invasive marker of sepsis or shock progression and treatment response due to the return of flow following resuscitation.

## Discussion

Sepsis is a life-threatening condition with high mortality risk without prompt recognition and treatment. Currently, there is no validated, reproducible method to measure the microcirculation in sepsis. OCTA can potentially monitor haemodynamic resuscitation by targeting good capillary perfusion. In an ideal world of precision medicine, such targeted therapy has the potential to improve outcomes in sepsis and other low-flow states. Several studies have demonstrated retinal blood flow changes in systemic illnesses, including sepsis, demonstrating the potential for OCTA to provide microcirculatory monitoring. When considering this with the studies that demonstrate retinal blood flow returns to baseline in septic models after resuscitation (41, 42), retinal blood flow could be used as a marker for sepsis progression and resuscitation. Further, these imaging techniques used were non-invasive and fast, so they have minimal impact on patients. Further studies on the responsiveness of retinal blood flow following resuscitation in septic patients are essential, but preliminary studies are promising (38–40).

In addition to reporting retinal blood flow alterations in systemic disease (29, 30, 33–35), Kannenkeril et al. also indicate that OCTA changes are associated with disease progression in CKD (31), showing promise as a marker of disease severity in other systemic disorders affecting the microcirculation. Coppolino et al. (32) found an association between pre-dialysis retinal VD and patients who subsequently experienced intradialytic hypotension, demonstrating prognostic value and concluding that OCTA could stratify the short-term risk of this complication. OCTA flow density correlated with IDF conjunctival microcirculation in addition to haemodynamic measures during haemorrhagic shock and after resuscitation, suggesting that peripheral microcirculation as seen in the conjunctiva changed in association with central microcirculation as seen in the retina (43). Both measures could therefore serve as non-invasive markers of microcirculation during sepsis and critical illness, but this finding requires validation in human studies.

There are gaps in this area of research, with the main limitation of small sample sizes in human studies. Different retinal blood flow metrics were used in each study, which makes comparison difficult, as well as different imaging techniques used and different OCTA manufacturers between studies. It would also be beneficial to measure OCT and OCTA sequentially in systemically ill and septic patients to investigate the extent of perfusion dysfunction and its correlation with neurocognitive dysfunction. Future studies would benefit from larger cohorts of patients, and it would be of value to include patients in different severities of sepsis to investigate how retinal perfusion may be different.

## Conclusion

Using OCTA to visualise retinal blood flow is a promising method of monitoring microcirculation during sepsis and response to resuscitation. Further human studies are warranted to determine how this could be implemented in a cohort of septic patients.

## Author contributions

EC was a major contributor in writing the manuscript. All authors read, edited and approved the final manuscript.

## Abbreviations

qSOFA: quick Sequential Organ Failure Assessment
BP: blood pressure
SOFA: Sequential Organ Failure Assessment
ITU: intensive therapy unit
OCT: optical coherence tomography
OCTA: optical coherence tomography angiography
MAP: mean arterial pressure
VD: vessel density
AF: atrial fibrillation
CKD: chronic kidney disease
CRAE: central retinal arteriolar equivalent
CRVE: central retinal venular equivalent
FAZ: foveal avascular zone
COPD: chronic obstructive pulmonary disorder
pO_2_: partial pressure of oxygen
FA: fluorescein angiography
RAFT: retinal arterial filling time
CI: cardiac index
IDF: incident dark field
BFI: blood flow index.
